# Does Attending to Information in Working Memory Improve Long-Term Memory?

**DOI:** 10.5334/joc.513

**Published:** 2026-08-11

**Authors:** Caro Hautekiet, Klaus Oberauer

**Affiliations:** 1 Vrije Universiteit Amsterdam, the Netherlands; 2 University of Zurich, Switzerland

**Keywords:** Attention, Working Memory, Long-Term Memory

## Abstract

There is broad agreement among memory researchers that WM processes can influence LTM formation. Prior research has suggested that holding information in WM for a longer time may benefit LTM because of mechanisms such as refreshing, elaboration, or consolidation – processes presumed to rely on focused attention. However, studies manipulating free time do not isolate attention effects from other factors. Several studies have used a more direct way to study the effect of attention on LTM performance by using prioritization techniques (such as retro-cuing and rewarding) whereby attention is directed towards specific information in WM during the WM retention interval. Here we review these studies. The results are mixed: Whereas some studies find LTM benefits for prioritized items, others do not. Variability in outcomes could not be explained by differences in memory set size, presentation mode, material type, kind of priority signal, or test format. When an LTM benefit is present, the difference in performance remains modest. Thus, focused attention in WM generates at best a small benefit to LTM. Most of the included studies made use of a surprise LTM test and thus, further research is needed to determine whether the intent to remember information in the long term interacts with attention to promote LTM encoding. Altogether, our review findings suggest that attention to information in WM is insufficient on its own for robust LTM encoding.

In many day-to-day situations, we rely on working memory (WM) to keep information temporarily available for use in an ongoing task. In some situations, we might need this information for later recall. In this case, we can rely on long-term memory (LTM). Some theoretical models view WM and LTM as separate *stores* (e.g., R. C. Atkinson & Shiffrin, 1968; Baddeley, 2012; Barrouillet & Camos, 2015), whereas others do not make this strict distinction (e.g., Cowan, 1995; Melton, 1963; Nairne, 1990; Oberauer, 2002). While these different theoretical models might not agree on how WM and LTM are related, they do agree that there is cooperation between these two. In line with this, several studies have demonstrated that processes on information in WM affect how well this information is remembered in the long term (e.g., Bartsch et al., 2018; Bartsch & Oberauer, 2021; Camos & Portrat, 2015; Cotton & Ricker, 2021; Craik & Tulving, 1975; Johnson et al., 2002; Labaronne et al., 2024; Loaiza & Lavilla, 2021; Loaiza & McCabe, 2012; Sabo & Schneider, 2025; Souza & Oberauer, 2017; see also Bartsch et al., 2024). However, it remains unclear what is happening with representations in WM to improve their performance in a delayed test.

Hartshorne and Makovski (2019) recently reviewed the literature on how working memory maintenance affects long-term memory. The authors ran a meta-analysis on 61 experiments from 31 articles and showed that, indeed, longer WM maintenance improves LTM compared to shorter maintenance. This conclusion was confirmed by 11 new experiments, for which the overall pattern was consistent with a small but reliable effect of WM maintenance duration on LTM performance. There are different hypotheses of what might happen during maintenance in WM to result in improved LTM performance. Some researchers argue that more time to refresh representations in WM results in better LTM performance (e.g., Camos & Portrat, 2015; Jarjat et al., 2018; Loaiza & McCabe, 2012; McCabe, 2008), whereas others suggest that people use this time for elaboration (e.g., Bartsch et al., 2018; Craik & Tulving, 1975; Loaiza & Lavilla, 2021). Another explanation is that this free time enables consolidation of the representation, leading to a more durable representation that can withstand decay and interference not only in WM but also in LTM (e.g., Cotton & Ricker, 2021; Labaronne et al., 2023). Despite differing in the specifics, for all three processes, researchers assume that they rely on focused attention within WM. This shows broad agreement about the pivotal role of attention in facilitating the transfer of information from WM to LTM.

However, varying WM maintenance duration to enable refreshing, elaboration, or consolidation is not ideal to isolate the effect of focused attention on the formation of LTM. We can only assume that WM representations undergo refreshing, elaboration, or consolidation during maintenance. There is no way to verify these assumptions independently of the effect of free time on memory.

Studies that use a more direct approach to manipulate focused attention in WM could offer a clearer view of the role of attention in making long-term memories. Such studies employ instructed prioritization techniques to direct attention to specific items during the retention interval by indicating which item is (most likely) to be tested (*retro-cue*) or which item will be worth more points when tested and responded to correctly (*reward*). For both prioritization modes, the cued or highly-rewarded item is assumed to be brought into the focus of attention. Evidence for this assumption comes from the finding of improved memory for the prioritized item compared to other, not-prioritized items in a test of WM (for reviews, see Hitch et al., 2020, on reward-based prioritization; Souza & Oberauer, 2016, on cue-based prioritization). Using these methods, participants are instructed to direct attention to a specific item within WM, which allows for a more controlled investigation of how focused attention within WM influences subsequent LTM performance.

If LTM performance relies on focused attention during WM retention, improved LTM performance should be observed for prioritized representations in WM compared to unprioritized representations in WM. The evidence on this prediction is mixed. Therefore, in an attempt to get a better understanding of the relationship between WM and LTM, this review serves to evaluate the existing literature on the effect of focused attention in WM on LTM. We will outline and compare the similarities and differences of studies with a suitable task setup, meaning studies with a WM task followed by a filler task and then a LTM test of the materials of the WM task, including one of the commonly used prioritization strategies and a means to check for a benefit of attention in WM.

## The consequences of focused attention on long-term memory performance

To review the literature, we sought behavioral experiments in which focused attention was manipulated using prioritization signals (such as a retro-cue) and in which both WM and LTM performance was measured in young healthy adults. We searched for articles using keyword searches (using words such as *prioritization, focused attention, working memory, long-term memory,…*) and by following citations forward and backward of already-identified papers (similar to the approach used by Hartshorne & Makovski, 2019). Using this procedure, we identified 15 articles (A. L. Atkinson et al., 2024; Born & Spitzer, 2024; Fan & Turk-Browne, 2013; Hartshorne & Makovski, 2019; Higgins et al., 2020; Jeanneret et al., 2023; M. K. Johnson et al., 2002; M. R. Johnson et al., 2013; LaRocque et al., 2015; Lintz & Johnson, 2021; Chao et al., 2024; Reaves et al., 2016; Sandry et al., 2020; Strunk et al., 2019; Wang & van Ede, 2024).^1^ We excluded studies that did not observe a performance benefit for the prioritized item in the WM test (Higgins et al., 2020; M. K. Johnson et al., 2002; M. R. Johnson et al., 2013) and studies in which the setup did not allow for measuring the benefits of prioritization in the WM test (Fan & Turk-Browne, 2013), because, for these studies, it is not clear whether the prioritization manipulation worked and thus, whether participants actually focused attention on the to-be-prioritized information.

After excluding these articles, 11 relevant studies remained. For some of these studies, we excluded experiments that were not suitable for comparison for the purpose of the current review. In particular, we excluded Experiment 1 by LaRocque et al. (2015) because this experiment focused on WM performance and did not include an LTM test. Hartshorne and Makovski (2019) conducted two experiments using a retro-cue (Experiments 12 and 13), the other experiments did not include a retro-cue or any other prioritization strategy and were therefore excluded. The same applies to Experiment 1 by Born and Spitzer (2024) and Experiment 2 by Sandry et al. (2020). In Experiment 1 by Lintz and Johnson (2021) and Experiment 1 by Wang and van Ede (2024), only validly cued items were tested in the working memory task and thus, this task set-up did not allow to check for a WM benefit. These experiments were therefore excluded. Furthermore, we only included Experiments 1 and 3 from Chao et al. (2024), because Experiment 2 included a divided attention task in between the cue and delay periods which complicates comparison to the other included studies. From the study by Jeanneret et al. (2023), the reward condition from Experiment 1 was excluded from our comparison, because the authors did not observe a WM benefit in this condition.

Table 1 presents an overview of the potentially theoretically relevant methodological differences and similarities between the included studies (see Table S1 in supplementary materials for other methodological differences and similarities). All these studies followed the same sequence of phases: WM task – filler task – LTM task. The LTM test was always presented at the end of the experiment. In the WM task, one of the to-be-memorized items was prioritized and thus assumed to be in the focus of attention during the retention interval of the WM task. In the LTM task, memory for this prioritized item is then compared to other, unprioritized items from the WM task. An important confound that should be taken into account in this type of task setup is whether or not the memory item was tested in the WM task (for a review on testing effects, see Rowland, 2014). Some of the studies in Table 1 did not take into account testing effects (see Table 1, “(1) regardless of testing in WM”). To measure the effect of WM prioritization on LTM performance, over and above potential effects of testing, we can consider two comparisons. First, we can compare LTM performance for the prioritized and unprioritized items that were both tested in the WM task (see Table 1, “(2) Both tested in WM”). In this comparison, LTM of both items would be similarly affected by being tested, but one was attended during the retention interval of the WM task whereas the other one was not. Second, the testing effect can be eliminated by including trials in which the memory test is omitted (see Table 1, “(3) Neither tested in WM”). In this way, one can compare LTM performance for the prioritized-untested items with that of the unprioritized-untested items. In this case, neither items were tested in WM, but one was attended during the retention interval of the WM task, whereas the other one was not. As it remains unclear whether testing in WM has a similar effect on the unprioritized item as on the prioritized item, the second approach is the most ideal to examine the isolated effect of attention in WM on LTM performance. Still, we will use both approaches to review the findings in the literature. When the results within one study contradict each other when taking in account testing effects or not, we will only focus on the results where testing has been taken into account.

**Table 1:** Overview of the relevant studies and results in terms of LTM benefit of attentional prioritization (with or without taking into account the testing effect in WM).


STUDY & EXPERIMENT		WM ITEMS				PRIORITY INFORMATION			MEMORY TESTS		LTM COMPARISON (PRIORITIZED VS. …)	LTM BENEFIT		
					
		SET SIZE	PRESENTATION	DURATION (PER ITEM, IN MS)	TYPE	TYPE	TIMING (RELATIVE TO ENCODING)	DURATION + POST DELAY (IN MS)	WM	LTM (E = EXPECTED, U = UNEXPECTED)		(1) REGARDLESS OF TESTING IN WM	(2) BOTH TESTED IN WM	(3) NEITHER TESTED IN WM

LaRocque et al. (2015)	2	2	Simultaneous	1000	Visual	Retro-cue	After	500 + 2000	Recognition	Recognition (U)	Uncued	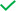	–	( 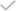 )
	
	3	2	Simultaneous	1000	Visual	Retro-cue	After	500 + 2000	Recognition	Recognition (U)		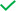	–	( 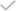 )

Reaves et al. (2016)	-	2	Simultaneous	100	Visual	Retro-cue	After	200 + 700–1100	Recognition	Recognition (E)	Neutral-cued	–	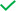	–

Strunk et al. (2019)	-	2	Simultaneous	100	Visual	Retro-cue	After	200 + 700–1100	Recognition	Recognition (E)	Neutral-cued	–	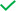	–

Hartshorne and Makovski (2019)	12	3	Simultaneous	770	Verbal	Retro-cue	After	4000 + 0	Recognition	Recognition (U)	Neutral-cued	–	–	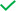 *
	
	13	4	Simultaneous	770	Visual	Retro-cue	After	4000 + 0	Recognition	Recognition (U)		–	–	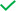 *

Sandry et al. (2020)	1	3	Sequential	500	Verbal	Reward	During	500 + 500	Recognition	Free recall (U)	Uncued and Neutral-cued	–	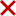	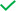

Lintz and Johnson (2021)	2	3	Simultaneous	750	Verbal	Retro-cue	After	1500 + 100	LDT	Recognition (U)	Uncued	–	–	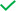

Chao et al. (2024)	1	2	Simultaneous	1000	Visual	Retro-cue	After	1000 + 2000	Recognition	Recognition (E)	Uncued	–	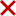	–
	
	3	2	Simultaneous	1000	Visual	Retro-cue	After	1000 + 2000	Recognition	Recognition (U)		–	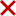	–

Jeanneret et al. (2023)	1	4	Simultaneous	250	Visual	Retro-cue	After	500 + 500	Recognition	Recognition (U)	Neutral-cued, Uncued	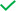	–	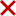
	
	2a	4	Simultaneous	250	Visual	Retro-cue	After	500 + 500	Recognition	Recognition (U)		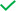	–	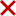

	2b	4	Simultaneous	250	Visual	Reward	After	500 + 500	Recognition	Recognition (U)	Low reward	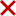	–	–

Atkinson et al. (2024)	1	4	Sequential	250	Visual	Reward	Before	250 + 250	Recognition	Recognition (U)	Low reward, Equal reward	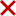	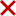	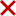
	
	2	4	Sequential	500	Visual	Reward	Before	500 + 250	Recognition	Recognition (U)		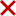	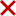	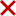

Wang and van Ede (2024)	2	2	Simultaneous	250	Visual	Retro-cue	After	500 + 1500	Recognition	Recognition (U)	Uncued and Neutral-cued	–	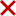	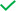 *

Born and Spitzer (2024)	2	2	Sequential	1500	Visual	Retro-cue	After	1000 + 4000	Reproduction	Reproduction (U)	Uncued	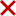	–	–


*Note.* WM = working memory, LTM = long-term memory, LDT = lexical decision task. A checkmark ‘
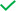
’ means statistical evidence for an LTM benefit, ‘
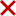
’ means no statistical evidence, and ‘(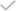)’ refers to a descriptive benefit. *These studies include items that were tested but there was no re-exposure of the items on screen (no-match trials). All studies above the thick black line were included in the meta-analysis.

We also collected accuracy data (number of correct trials and total number of trials) from the studies included in Table 1 for a small meta-analysis. We computed logistic models in R using the brms package (Bürkner, 2017) to estimate the evidence for or against an interaction between the attentional benefit on LTM and the different variables included in Table 1. The experiment by Born and Spitzer (2024) was not included in this analysis because they reported recall error rather than proportion correct. For the included studies, where possible, we selected the results of untested trials (A. L. Atkinson et al., 2024; Hartshorne & Makovski, 2019; Jeanneret et al., 2023; LaRocque et al., 2015; Lintz & Johnson, 2021; Sandry et al., 2020; Wang & van Ede, 2024).^2^ If this was not possible, we selected the results of trials where both prioritized and unprioritized items had been tested (Chao et al., 2024; Reaves et al., 2016; Strunk et al., 2019). We started from the full model including the main effect of Priority Status (prioritized vs. unprioiritized in WM) and the interactions between Priority Status and (1) Set Size, (2) Presentation Mode, (3) Encoding Duration, (4) Memory Materials, (5) Priority Type, (6) Priority Timing, (7) Priority Duration, and (8) LTM Test Awareness. Next, we used a hierarchical approach to evaluate the main effect of Priority Status on LTM performance, and its interactions with the eight potential moderators. We started comparing the full model to a model excluding the interaction between Priority Status and each of the other variables in the order listed above. In case there was evidence in favor of this interaction (BF > 3), we continued with a model including this interaction, otherwise we continued with a model excluding this interaction. We proceeded in this way until we had obtained evidence for or against all interactions and the main effect of Priority Status. We will discuss each of the included variables and the results of our analysis in the sections below.

## Variations in the Presentation of Working Memory Items

The studies included in Table 1 made use of different WM set sizes, ranging from two to four items. While there is no clear pattern in the results, it seems that studies using smaller set sizes are more likely to detect an LTM benefit (LaRocque et al., 2015; Lintz & Johnson, 2021; Reaves et al., 2016; Sandry et al., 2020; Strunk et al., 2019; Wang & van Ede, 2024). Still, we observed evidence against an interaction between Set Size and the Priority Status of the items in our logistic models (BF_10_ = 0.16). Thus, the differences in set sizes cannot explain the differences in results presented in Table 1.

The studies included in Table 1 made use of different item presentation modes, such that some studies presented the memory items simultaneously, whereas others presented them sequentially. Additionally, the encoding durations range from 100 ms – 1500 ms per item. Our analysis of the different studies showed inconclusive evidence for an interaction between Presentation Mode and the Priority Status of the items (BF_10_ = 1.13) and strong evidence against the interaction between Encoding Duration and Priority Status (BF_10_ = 0.09). Therefore, we cannot rule out presentation mode as a moderator. Encoding duration is unlikely to explain why some studies observed an LTM benefit of WM prioritization, whereas others did not.

Whereas most studies included in Table 1 used visual memory materials, there are three experiments included in which verbal materials were used. For all three of these experiments, an LTM benefit was obtained (Experiment 12, Hartshorne & Makovski, 2019; Lintz & Johnson, 2021; Sandry et al., 2020). Still, the results of our analysis over the different experiments showed inconclusive evidence against the interaction between the memorized materials and the priority status of the items (BF_10_ = 0.58). Thus, the difference in materials is unlikely to explain the difference in results.

## Variations of the Priority Signal

The studies included in Table 1 mostly used a retro-cue, which is typically a spatial cue presented during the retention interval of a WM task (after encoding) that indicates which item is most likely to-be-tested at the end of the trial (for a review, see Souza & Oberauer, 2016). In most of the included studies, the retro-cue even indicated the to-be-tested item with 100% validity (Born and Spitzer made use of a 75 or 83.33%-valid cue, and Lintz and Johnson made use of a 40%-valid cue). In four experiments, reward values were used to prioritize an item in WM (A. L. Atkinson et al., 2024; Jeanneret et al., 2023; Sandry et al., 2020). In these studies, one of the memory items was assigned a higher reward value when tested and responded to correctly compared to the other items (for a review, see Allen et al., 2024). In the included studies, the reward values could be assigned through a location cue after encoding (similar to the retro-cue) or could be linked to the serial position of the item. Given that the studies that observed an LTM benefit of WM-prioritization did not consistently use a retro-cue or reward values, the type of priority signal and its timing in the WM task can probably not explain the differences in obtained results. In line with this, our analysis resulted in inconclusive evidence against the interaction between the type of priority signal and the priority status of the items (BF_10_ = 0.55).

One could argue that the duration of the priority signal as well as the delay time following it could be critical, as more time for the item to reside in the focus of attention could result in improved LTM performance (e.g., Hartshorne & Makovski, 2019; Souza & Oberauer, 2017). Indeed, most of the studies that did observe an LTM benefit from WM prioritization presented the priority signal for a longer period and/or combined this with a longer post-priority signal delay, ranging from 1000 ms to 4000 ms (Hartshorne & Makovski, 2019; LaRocque et al., 2015; Lintz & Johnson, 2021; Reaves et al., 2016; Strunk et al., 2019; Wang & van Ede, 2024). In some of the studies that did not observe an LTM benefit, this duration was much shorter, ranging from 500 ms to 1000 ms (A. L. Atkinson et al., 2024; Jeanneret et al., 2023). Still, we observed very strong evidence against an interaction between the duration of the priority signal (including the delay time after) and the priority status of the items (BF_10_ = 0.02). Thus, the presentation rate of the priority signal, including the duration of the post-priority signal delay, cannot explain the different results.

## Variations in the Memory Tests

Another task parameter that could potentially explain the differences in results for the LTM benefit of WM prioritization is the type of WM and LTM test. For example, a more precise measure of memory performance (such as recall error of a reproduction test) might be more sensitive to detect an LTM benefit of WM prioritization than more blunt measures (such as accuracy from a recognition test). However, almost all studies made use of a recognition test for the WM task (Lintz and Johnson, 2021, used a lexical decision task, and Born and Spitzer, 2024, used a reproduction test). Thus, the type of WM task is unlikely to explain the obtained difference in LTM results. Almost all studies also used a recognition test for the LTM task (Sandry et al., 2020, used free recall, and Born and Spitzer, 2024, used a reproduction test).^3^ Thus, the type of LTM test is unlikely to explain the differences in results.

Furthermore, the intent to remember could potentially make a difference in whether or not a WM prioritization benefit can be observed in LTM performance. From the studies included in Table 1, the LTM test was announced beforehand, and thus expected, in only three experiments (Experiment 1, Chao et al., 2024; Reaves et al., 2016; Strunk et al., 2019). Whereas Reaves et al. (2016) and Strunk et al. (2019) did observe an LTM benefit of WM prioritization, Chao et al. (2024) did not. Chao et al. (2024) even compared two experiments in which participants were either aware (Experiment 1) or unaware (Experiment 3) of the upcoming LTM test, but neither of the experiments resulted in an LTM benefit of WM prioritization. Our analysis resulted in inconclusive evidence against an interaction between LTM test awareness and the priority status of the items (BF_10_ = 0.38). Thus, based on these studies, the intent to remember is not a compelling explanation of the difference in results. Still, it seems plausible that people might be more efficient in using attention in WM to benefit LTM when they are aware of the upcoming LTM test compared to when they are unaware (see also Popov & Dames, 2023). This possibility will be further addressed in the General Discussion.

## Variations in the Long-Term Memory Comparison

A final variable that could be critical to explain the differences in obtained LTM results is the comparison that is made to measure the potential LTM benefit. Specifically, in the WM literature, the performance difference is often calculated by comparing performance for the prioritized items to performance for the neutral or baseline items, that is, items from a trial in which no priority signal was present. This comparison reflects the true performance benefit of prioritization. In contrast, one can also calculate the performance difference between the prioritized items and the unprioritized items from the same trials. This comparison reflects the combination of the performance benefit of prioritization as well as the performance cost for unprioritized items in trials with a priority signal. In the WM literature, some studies have already demonstrated that these costs and benefits are not equally large and that, on some occasions, one can be present without the other (e.g., Hautekiet et al., 2025; Vergauwe et al., 2025). Thus, it is possible that some of the LTM benefits observed in the studies included in Table 1 are actually a combination of the cost and the benefit, rather than reflecting a pure benefit.

When comparing the studies included in Table 1, it seems that most studies that did observe an LTM benefit did indeed compare the prioritized item to an unprioritized item from the same type of trials, and thus, the observed benefit might actually be a combination of the costs and benefits of prioritization rather than a pure benefit. However, some of the studies that did not obtain an LTM benefit also made use of the same comparison. Therefore, based on this, it seems that the differences in results cannot be explained by the prioritization comparison made to measure the LTM benefit.^4^

## Overall effect of attention in WM on LTM

As a final step of our meta-analysis, we also looked at the overall evidence for an effect of attention in WM on LTM by comparing a model including the main effect of Priority Status, the random effects and a random intercept to a null model, including the same random effects structure. Taking into account all relevant studies, we observed inconclusive evidence against a main effect of Priority Status (BF_10_ = 0.36).

## Discussion

The question asked in this review is whether focused attention in WM is beneficial for the transfer of information from WM to LTM. We compared experiments from 11 articles that used a similar task setup to review the evidence in the literature. Overall, the results in the literature are mixed. When taking into account testing effects in WM, about half of the experiments detected an LTM benefit for WM prioritization. When comparing studies that did vs. did not observe LTM benefits of WM prioritization, no clear pattern emerged. A meta-analysis of the relevant studies also showed that none of the identified task parameters can convincingly explain the differences in obtained results. The inconsistencies in the results, as well as the fact that there is no clear pattern to explain these differences, raises the question as to whether attention in WM is actually beneficial for LTM performance or not.

A closer examination of the studies that did observe an LTM benefit of WM prioritization shows that the effect reflects a rather modest difference in performance. For example, Hartshorne and Makovski (2019) observed a difference of about 8% (Experiment 12) and 3% (Experiment 13) accuracy between the cued and uncued items. Sandry et al. (2020) observed a difference of about 0.004 in the proportion of items retained between high and low reward items (Cohen’s *d* = 0.33). Although supported by a non-negligible Bayes factor (BF = 4.05), it is a small difference in performance. Lintz and Johnson (2021) observed a difference of 0.2 in confidence rating between cued and uncued items (Cohen’s *d* = 0.755). Similarly, Wang and van Ede (2024) observed a difference of about 6% in accuracy between the cued and uncued/neutral cued items on the LTM test. Thus, while these studies did observe an LTM benefit for WM prioritization, the difference in performance in LTM for prioritized and unprioritized items remains rather modest. If the LTM benefit that can be obtained from WM prioritization is indeed a small effect, this could explain why it cannot consistently be observed in the literature, and why the evidence pooled over all studies is leaning against a main effect.

Does this mean that attention is not needed to create long-term memories? This conclusion would be premature. Both prioritized and unprioritized items (from cue trials and no-cue trials) have been encoded into WM, and presumably, that involved some degree of attention to them when they were presented. What the findings from our review suggest is that directing additional focused attention to items once they are already in WM has at best a small additional effect on long-term retention of these items.

Perhaps an initial brief moment of attention to a stimulus is enough to establish it in LTM, and further attention to it does not yield any further benefit? That does not seem to be the case, because both extension of study time and repeated presentation of stimuli for study are well-established experimental techniques for strengthening individual items in LTM. However, in these cases, the person has the intention to encode the stimulus for a subsequent test of LTM. This was not the case in most of the studies reviewed here: When a prioritization signal directed attention to one item in WM, participants usually did not anticipate that this item would be relevant beyond the duration of the WM-task trial. In the present review, we found only three studies in which participants were expecting the LTM test, and in two of them, prioritization had a beneficial effect on LTM. Therefore, we should consider the following possibility: Initial attention to a stimulus for about 200 to 1000 ms – the typical presentation duration of stimuli in a WM test – generates a trace in LTM independently of the person’s intention to remember it. This is the basis of incidental memory for attended events. Attending to the same stimulus again – either in the perceived environment or in WM – does not add much to its strength or accessibility in LTM. However, when such a second period of attention to the stimulus is accompanied by processes suited for generating strong LTM representations – such as elaboration – then the second attention period is likely to yield a more substantial benefit in LTM. Participants are more likely to employ such processes when they are aware of an upcoming LTM test and therefore try to establish good LTM representations. This could explain why repeated presentation as well as longer presentation time are effective means to strengthen memory in intentional-memory studies, while they are less effective in incidental-memory studies (e.g., Helbing et al., 2020; Tatler & Tatler, 2013).

While not the main focus of our review, the studies included in Table 1 also demonstrated mixed findings in terms of the effect of testing in WM on LTM performance. While some studies did observe an effect of testing on LTM performance (e.g., A. L. Atkinson et al., 2024; Jeanneret et al., 2023), others did not (e.g., Hartshorne & Makovski, 2019; Sandry et al., 2020). In our comparison of Table 1, we followed authors’ descriptions of tested vs. not tested WM items, but some of the studies created a “no-test” condition by eliminating re-exposure to the correct stimulus at test rather than actually removing the memory test (A. L. Atkinson et al., 2024; Hartshorne & Makovski, 2019; Wang & van Ede, 2024). Thus, in these experiments, participants still had to compare the target item in mind to the probes on screen, even if the actual target was not represented on screen. It is possible that some of the processes involved with testing are still present when comparing the target item to the probe, resulting in a benefit for LTM. If we would – for that reason – take out these studies from our comparison, even less evidence would remain for the benefit of focused attention in WM on LTM performance.

In summary, our review and meta-analysis of the relevant literature demonstrates that – when participants are unaware of the upcoming LTM test – there is little evidence that focused attention in WM benefits LTM. This means that attention might be a necessary but not sufficient condition for LTM learning. Additional processes that depend on the intention to learn might contribute. To better understand the role of attention in creating long-term memories, future studies should further examine whether focused attention in WM benefits LTM when there is an intent to remember, and what the difference is between intentional and incidental memorization.

## Additional File

The additional file for this article can be found as follows:

10.5334/joc.513.s1 Supplementary Materials. Additional information reviewed studies.

## Footnotes

There are also studies in the literature that examined the effect of focused attention on already-encoded LTM information (e.g., Gong et al., 2025; Sabo & Schneider, 2025). However, in this review, we focused on how attention benefits the transfer of information from WM to LTM, rather than the bidirectional relationship between WM and LTM.

There was a slight discrepancy between the published results and the results we reproduced from the published data for the study by Lintz and Johnson (2021). Despite the authors’ cooperation, this could not be fully resolved; however, because the discrepancy was minor and the authors confirmed the published data are valid, the study was included. For this study, confidence ratings were recoded to accuracy scores, with “definitely no” and “maybe no” coded as “non-match”, and “maybe yes” and “definitely yes” coded as “match”.

This was not included as a variable in our analysis because there was only one study that used a different WM test other than recognition (Lintz & Johnson, 2021) and only one study that used a different LTM test, again other than recognition (Sandry et al., 2020).

We did not include this variable in the analysis because some studies compared to both uncued or low-reward items as well as to neutrally-cued or equally-rewarded items. For the analysis, where possible, we always selected the data from neutral trials because this reflects the attentional benefit more genuinely than when it also includes a potential cost from comparison to uncued/low-reward trials.
