# Supplementary material for: Does Attending to Information in Working Memory Improve Long-Term Memory?
Source: J Cogn. 2026 Aug 11;9(1):41. doi: 10.5334/joc.513 (PMC13475634; doi:10.5334/joc.513)
Supplement: Supplementary file 1 [file joc-9-1-513-s1.pdf]

Supplementary Materials: Additional information reviewed studies

Additional information on the experimental design and the data collection procedure of the reviewed studies can be found in Table S1. Information about the design, number of trials, and trial division can be found below the table. More details on the methods and procedures can be found in each of the respective articles.

Table S1

Additional information for the studies included in Table 1.

Study & Experiment		Experimental design				Data collection		LTM benefit			
		Randomization	Time between WM-LTM	Articulatory suppression	Feedback WM task	Sample size	Format	Regardless of testing	Both tested	Neither tested	Effect sizes
LaRocque et al. (2015)	2	Randomly intermixed	7-8 days	No#	Yes	17	In person#	✓	-	(✓)	Not reported
	3	Randomly intermixed	40 min.	No#	Yes	16	In person#	✓	-	(✓)	Not reported
Reaves et al. (2016)	-	Randomly intermixed	25 min.	No#	No#	19	In person#	-	✓	-	Not reported
Strunk et al. (2019)	-	Randomly intermixed	25 min.	No#	No#	25	In person#	-	✓	-	$\eta_p^2 = 0.33$
Hartshorne and Makovski (2019)	12	Randomly intermixed	Several min.	No#	No#	342	Online	-	-	✓	Not reported
	13	Randomly intermixed	Several min.	No#	No#	472	Online	-	-	✓	Not reported
Sandry et al. (2020)	1	Randomly intermixed	3 min.	Yes	Yes	67	In person#	-	✗ [1]	✓ [2]	(1) $\eta_p^2 = 0.008$ (2) d = 0.33
Lintz and Johnson (2021)	2	Randomly intermixed	10 min.	No#	No#	23	In person#	-	-	✓	*d* = 0.477
Chao et al. (2023)	1	Randomly intermixed	5 min.	No#	No#	30	Both	-	✗	-	$\eta_p^2 = 0.045$
	3	Randomly intermixed	5 min.	No#	No#	33	Online	-	✗	-	$\eta_p^2 = 0.128$
Jeanneret et al. (2023)	1	Blocked	2 min.	No	No#	27	In person	✓	-	✗	Not reported
	2a	Blocked	2 min.	No	No#	32	In person	✓	-	✗	Not reported
	2b	Blocked	2 min.	No	No#	32	In person	✗	-	-	Not reported
Atkinson et al. (2024)	1	Randomly intermixed	10 min.	Yes	No#	24	In person#	✗	✗	✗	$\eta_p^2 < .01$
	2	Randomly intermixed	10 min.	Yes	No#	24	In person#	✗	✗	✗	$\eta_p^2 = .01$
Wang and van Ede (2024)	2	Randomly intermixed	10 min.	No#	Yes	50	In person	-	✗	✓	Not reported
Born and Spitzer (2024)	2	Randomly intermixed	1 min.	No#	No#	89	Online	✗	-	-	$\eta^2 = 0.0005$

Note. WM = working memory, LTM = long-term memory, LDT = lexical decision task. A checkmark '✓' means statistical evidence for an LTM benefit, '✗' means no statistical evidence, and '(✓)' refers to a descriptive benefit. Sample size represents the sample after exclusion. More information about trial division is included in Section 3. All information marked with "#" was not explicitly reported in the article.
